# Testing practices and clinical management of lipoprotein(a) levels: A 5-year retrospective analysis from the Johns Hopkins Hospital

**DOI:** 10.1016/j.ajpc.2024.100686

**Published:** 2024-06-19

**Authors:** Yehuda Eidensohn, Anjali Bhatla, Jie Ding, Roger S. Blumenthal, Seth S. Martin, Francoise A. Marvel

**Affiliations:** a Ciccarone Center for the Prevention of Cardiovascular Disease, Division of Cardiology, Department of Medicine, Johns Hopkins University School of Medicine, Baltimore, Maryland, United States; b Division of Cardiology, Department of Medicine, Johns Hopkins University, Baltimore, Maryland, United States

**Keywords:** Cardiovascular disease, Lipoprotein(a), Cholesterol, Lipid, Primary prevention, Secondary prevention, Screening

## Abstract

**Objective:**

Elevated lipoprotein(a) [Lp(a)] is an independent, genetically determined risk factor for atherosclerotic cardiovascular disease (ASCVD). We evaluated the frequency of testing for elevated Lp(a) and subsequent management at the Johns Hopkins Hospital, a large academic medical center, over a 5-year period.

**Methods:**

The Johns Hopkins Hospital (JHH) electronic medical record was queried to identify patients with an encounter between 2017 and 2021, either with established ASCVD or at increased risk, defined as being on any lipid lowering medication or having LDL-C ≥ 190 mg/dL. The frequency of Lp(a) testing and of elevated levels were identified for each year.

**Results:**

Among 111,350 unique adult patients, 2,785 (2.5 %) had at least one Lp(a) test. Patients with Lp(a) testing, compared to those without testing, were younger (mean age 56 years vs. 66 years), more often female (49 % vs. 44 %), Black (24.7 % vs. 24.6 %) or “other” race/ethnicity (12 % vs 10 %), and had higher LDL-C levels (median 118 vs. 91 mg/dL; *p* < 0.001). The number and frequency of Lp(a) testing increased from 167 (0.57 %) in 2017 to 1155 (5.67 %) in 2021. Lp(a) levels were abnormal in 43.4 % of patients (moderate [75–125 nmol/L]: 10.3 %, high [126–600 nmol/L]: 32.2 %, severe [>600 nmol/L]: 0.9 %). Among 920 patients with high or severe Lp(a) levels, 200 (22 %) had a subsequent referral to cardiology or lipid specialist, and 180 (20 %) had a new lipid-lowering medication prescribed in the subsequent 18 months.

**Conclusion:**

Based on a single-center experience, the frequency of incident Lp(a) testing among increased-risk patients was low but increased significantly over 5-years, likely due to Lipid Clinic referrals with reflex Lp(a) testing and greater awareness about this risk factor. Future work should target appropriate population based Lp(a) testing strategies and clinical decision-making regarding risk management once Lp(a) elevation is diagnosed.

## 1 Introduction

Elevated lipoprotein(a) [Lp(a)] is an independent, causal, and genetically determined risk factor for atherosclerotic cardiovascular diseases (ASCVD) and calcific aortic stenosis due to increased inflammation and atherogenesis [1]. Lp(a) levels >125 nmol/L or ≥ 50 mg/dL are associated with 50–60 % increase in risk of ASCVD, [[2], [3], [4], [5], [6]] and risk increases at higher levels in a linear fashion. Approximately 20–30 % of the population has elevated Lp(a) [7]. When combined with elevated LDL-C ≥ 130 mg/dL, elevated Lp(a) confers greater risk for cardiovascular events, and even when medications are utilized to lower LDL-C, individuals with elevated Lp(a) have a 10 % residual risk for recurrent events [8]. Given this, the AHA/ACC 2018 Guidelines on the Management of Blood Cholesterol identified elevated Lp(a) as a risk-enhancing factor favoring more intensive management of lipids [9]. Despite the established contribution of elevated Lp(a) on ASCVD, Lp(a) testing remains low due to (1) clinician awareness and education, (2) insufficient access to cardiology and Lipid Clinic specialists, (3) lack of standardization of testing assays, and the (4) lack of approved medications that directly target Lp(a) levels to mitigate ASCVD risk [10].

Prior single institution studies[[11], [12], [13], [14]] have shown that Lp(a) testing is low; however, these studies did not focus on the subgroup of increased-risk and secondary prevention subpopulations, where the clinical utilization of elevated Lp(a) diagnosis may have a greater impact in decreasing ASCVD events by informing clinical decision-making regarding risk management. In addition, these studies did not evaluate the clinical decision-making that resulted from Lp(a) testing, including lipid lowering medication management and referrals to cardiology and/or lipid clinic. We aimed to quantify Lp(a) testing practices in patients at increased risk of ASCVD at a large academic medical center.

## 2 Methods

This was a single center, retrospective cohort study conducted at the Johns Hopkins Hospital (JHH) and outpatient center in 2023. The JHH electronic medical record was queried to identify patients aged 18 and over with an encounter over the 5 year period between 2017 and 2021 who had one of the following increased risk features: 1) A low density lipoprotein-calculated (LDL-C) level ≥ 190 mg/dL; 2) Established cardiovascular, cerebrovascular, or peripheral arterial disease, as determined by ICD-10 and CPT codes; 3) Prescribed lipid-lowering medication(s), including statins, fibrates, ezetimibe, bempedoic acid, and PCSK9 inhibitors. The study was approved by the Johns Hopkins Medicine Institutional Review Board.

Although European guidelines currently recommend universal screening of Lp(a), the scope of this study was limited to patients most likely to benefit from screening and subsequent interventions. Our increased-risk group was designed to mimic National Lipid Association (NLA), American Association of Clinical Endocrinologists/ American College of Endocrinology, and American College of Cardiology/American Heart Association guidelines when to utilize Lp(a) [[7], [9], [15]]. For primary prevention, Lp(a) utilization is suggested in patients of borderline to intermediate 10-year risk (5 %–19.9 %) for ASCVD by the Pooled Cohort Equation. Being on LLT was used as a pragmatic indicator of at least borderline risk. The NLA recommends testing Lp(a) in all patients with primary severe hypercholesterolemia (LDL-C ≥ 190 mg/dL) or premature ASCVD, which the AACE/ACE extends to all patients with ASCVD [15,16].

The primary outcomes were the frequency of Lp(a) testing and the prevalence of high Lp(a). Lp(a) testing at JHH is performed at Quest using an immunoturbidimetric assay calibrated to the World Health Organization/International Federation of Clinical Chemistry reference material which is not affected by the number of Kringle repeats and is reported in the preferred units of nmol/L. Lp(a) levels (nmol/L) were defined as optimal (< 75), moderate (75–125), high (126–600) and severe (> 600). Lp(a) > 600 nmol/L is the upper limit of detection of our laboratory and given the consistent linear association between Lp(a) elevation and ASCVD risk, this category was added to the laboratory reported cutoffs of 75 and 125 nmol/L to identify extremely high-risk patients. “High” and “severe” groups were combined for most analyses and will be referred to collectively as “elevated” or “high” unless otherwise specified. Secondary outcomes included rates of referrals to cardiology or lipid clinic, prescriptions of lipid lowering medications, and LDL-C levels.

For patients with multiple Lp(a) or LDL-C values, the highest value was considered the index value. LDL-C values obtained after the index Lp(a) value were not used, given the fact that any lab results available post-Lp(a) testing would not have influenced the decision to order the Lp(a) test.

Descriptive statistics were conducted for the primary outcome variables and stratified by sex and age. Chi-square, *t*-test, and Mann-Whitney tests were used to compare the differences across patient characteristics. Subgroup analyses were conducted among those with premature ASCVD, defined as ages 18 to 55 years for males and 18 to 65 years for females. All statistical tests were two-sided. Data was analyzed using STATA version 18.0.

## 3 Results

A total of 111,350 patients were identified. Mean age (SD) at the index encounter was 65 (13) years; 49,065 (44.1 %) were female. 53,535 (48 %) had established ASCVD and 3358 (3 %) had severe aortic stenosis. 65,188 (58.5 %) were prescribed LLT, 1263 (1.13 %) had an LDL-C level ≥ 190 mg/dL, and 510 (0.5 %) had both an LDL-C level ≥ 190 mg/dL and were prescribed LLT. Baseline characteristics are presented in Table 1.

**Table 1:** Characteristics of Patients According to Lp(a) Testing Status.

	All patients	Patient with Lp(a) checked	Patient with Lp(a) not checked	p-value
N	111,350	2785	108,565	
Age at index visit or Lp(a) test, mean (SD), years	65.4 (13.1)	56.2 (14.0)	65.6 (13.0)	<0.001
Female sex	44.1 % (49,065)	48.7 % (1357)	43.9 % (47,708)	<0.001
Race/ethnicity				<0.001
White	65.5 % (72,970)	63.2 % (1761)	65.6 % (71,209)	
Black	24.8 % (27,655)	24.7 % (687)	24.6 % (26,968)	
Other	9.6 % (10,725)	12.1 % (337)	9.8 % (10,388)	
Diabetes	15.0 % (16,723)	14.9 % (414)	15.0 % (16,309)	0.82
Hypertension	20.9 % (23,316)	19.9 % (554)	21.0 % (22,762)	0.17
Smoking	0.3 % (316)	0.3 % (9)	0.3 % (307)	0.69
ASCVD	48.1 % (53,535)	60 % (1671)	47.8 % (16,864)	<0.001
Severe aortic stenosis	3.0 % (3358)	2.6 % (73)	3.0 % (3285)	0.22
On lipid lowering therapy	58.5 % (65,188)	66.0 % (1838)	58.4 % (63,350)	<0.001
LDL-C, median (interquartile range)	92 (67–124)	118 (84–158)	91(67–123)	<0.001
LDL-C level ≥ 190 mg/d	1.1 % (1263)	4.2 % (116)	1.1 % (1147)	<0.001

Abbreviations- Lp(a): lipoprotein(a); SD: standard deviation; ASCVD: atherosclerotic cardiovascular disease; LDL-C: low density lipoprotein cholesterol.

**Fig. 1:**
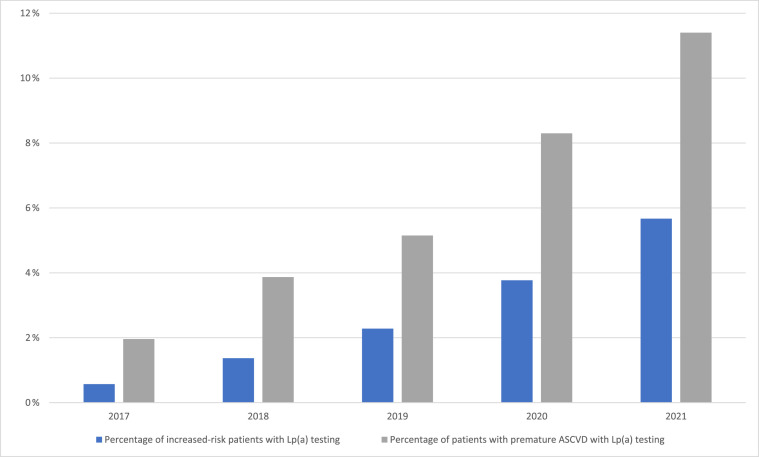
Frequency of Lp(a) Testing by Year

Among all patients, 2785 had Lp(a) checked during the study period. The number and frequency of testing increased from 167 (0.6 %) in 2017 to 1155 (5.7 %) in 2021, as shown in Fig. 1. Patients with Lp(a) testing, compared to those without testing, were younger (mean age 56 years vs. 66), more likely to be female (49 % vs. 44 %), Black (24.7 % vs. 24.6 %) or “other” race/ethnicity (12 % vs 10 %), more often had ASCVD (60 % vs 48 %), and had higher LDL-C (median 118 vs. 91 mg/dL; *p* < 0.001).

**Fig. 2:**
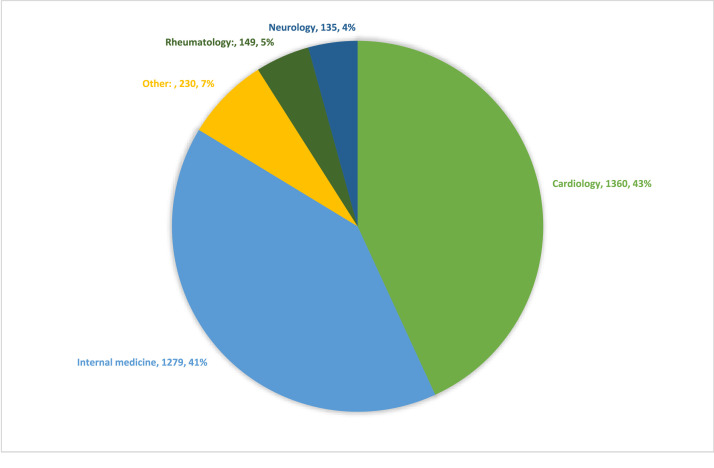
Lp(a) Ordering Specialty for the Years 2017–2021.

The most common ordering clinical specialty was cardiology (43 %), followed by internal medicine (41 %), as shown in Fig. 2. The percentage of Lp(a) tests ordered by cardiologists increased from 26 % of all tests in 2017 to 37 % in 2021. The percentage ordered by internal medicine declined from 43 % in 2017 to 38 % in 2021. Across specialists and generalists, rheumatology had the largest percentage change over the study period, increasing from 0.6 % of all tests in 2017 to 12 % in 2021.

**Fig. 3:**
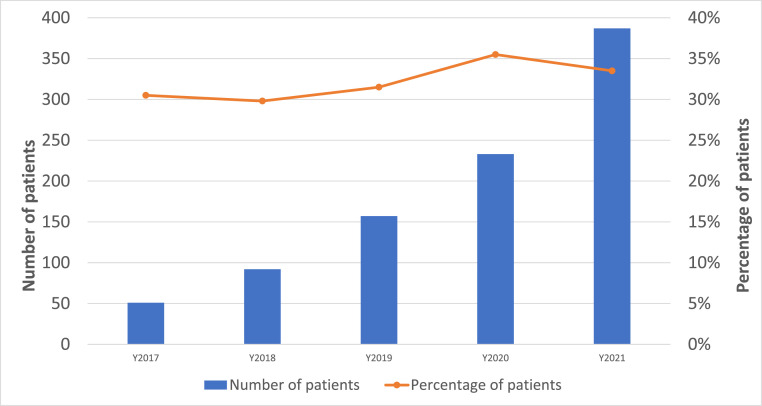
Frequency of Elevated Lp(a) by Year Among Patients with Lp(a) Testing (*N* = 2785).

Lp(a) levels were abnormal in 43 % of patients (moderate: 10 %, high: 32 %, severe: 0.9 %). Patients with high Lp(a) >125 nmol/L, compared to those without high Lp(a), were more likely to be female (55 % vs 45 %, *p* < 0.001), Black (42 % vs. 16 %, *p* < 0.001), have hypertension (22 % vs 19 %, *p* = 0.04), and had higher LDL-C (median 125 vs 114 mg/dL, *p* < 0.001), as shown in Table 2. Proportions with high Lp(a) among those tested increased from 31 % in 2017 to 36 % in 2020, as shown in Fig. 3. Among patients with established ASCVD, the proportion of high Lp(a) increased from 32 % in 2017 to 38 % in 2020, similar to the overall trend.

**Table 2:** Characteristics of Patients with Elevated Lp(a).

	Patients with high Lpa (>125 nmol/L)	Patient without high Lpa (<125 nmol/L)	p-value
N	920	1865	
Age at index visit or Lp(a) measurement, mean (SD), years	56.6 (13.6)	56.1 (14.2)	0.41
Female sex	55.4 % (510)	45.4 % (847)	<0.001
Race/ethnicity			<0.001
White	49.2 % (453)	70.1 % (1308)	
Blacks	42.1 % (387)	16.1 % (300)	
Other	8.7 % (80)	13.8 % (257)	
Diabetes	15.1 % (139)	14.8 % (275)	0.8
Hypertension	22.1 % (203)	18.8 % (351)	0.04
Smoking	0.4 % (4)	0.3 % (5)	0.47
ASCVD	62.1 % (571)	59.0 % (1100)	0.12
Severe aortic stenosis	2.7 % (25)	2.6 % (48)	0.82
On lipid lowering therapy	68.7 % (632)	64.7 % (1206)	0.04
LDL, median (interquartile range)	124.5 (92–163)	114 (81–155)	<0.001
LDL-C level ≥190 mg/dL	4.8 % (44)	3.9 % (72)	0.25

Abbreviations- Lp(a): lipoprotein(a); SD: standard deviation; ASCVD: atherosclerotic cardiovascular disease; LDL-C: low density lipoprotein cholesterol.

Among the 920 patients with Lp(a) levels > 125 nmol/L, 200 (22 %) had a subsequent referral to cardiology or lipid clinic. Notably, an additional 294 (32 %) either had a previous referral or their index Lp(a) test was ordered by a cardiologist. 61 % of the referrals were to general cardiology and 39 % were specifically for lipid clinic. The median time (interquartile range) between elevated Lp(a) and referrals was 21 days (4, 170.5). The absolute number of referrals increased from 16 in 2017 to 74 in 2021; however, the percentage of patients with elevated Lp(a) being referred declined from 31 % in 2017 to 19 % in 2021. Referrals were most frequently ordered by internal medicine (40 %) and cardiology (28 %). Among the 56 referrals placed by cardiologists, 38 (68 %) were to other cardiologists and 18 (32 %) were specifically for lipid clinic. Patients who received referrals, compared to those without referrals, were more likely to be black (57 % vs 38 %, *p* < 0.0001) and female (65 % vs 53 %, *p* = 0.003).

Among patients with Lp(a) levels > 125 nmol/L, 180 (20 %) had a subsequent newly prescribed lipid-lowering medication. The median time (interquartile range) between elevated Lp(a) and new prescriptions was 318 days (100.5, 521.5). The most common new medications were PCSK9 monoclonal antibodies (alirocumab and evolocumab), prescribed to 105 patients (58 %), followed by icosapent ethyl which was prescribed to 61 patients (34 %). Only 7 patients (4 %) had a new prescription of a statin, 4 patients (2 %) were prescribed bempedoic acid, and 2 patients (1 %) were prescribed ezetimibe. The absolute number of new medications increased from 10 in 2017 to 55 in 2021. Patients prescribed new medications, compared to those not prescribed new medications, were more likely to be white (67 % vs 45 %, *p* < 0.0001). There was no significant difference by gender (55 % female in both groups). No patients were referred for lipoprotein apheresis.

A separate analysis was conducted among patients with premature ASCVD, defined as ages 18 to 55 years for males and 18 to 65 years for females. In this subgroup of 14,534 patients, 851 (6 %) had an Lp(a) test. Rates of Lp(a) testing rose from 2 % in 2017 to 11 % in 2021. A total of 305 patients (36 % of those tested) had elevated Lp(a), of whom 30 % had a subsequent referral to cardiology or lipid clinic and 18 % had a subsequent prescription of a lipid lowering medication.

## 4 Discussion

In this study of Lp(a) testing practices in patients at increased risk of ASCVD at a large academic medical center, we found that Lp(a) testing is low but has been steadily increasing over time, with a nearly 10-fold increase (0.6 % to 5.6 %) in Lp(a) testing from 2017 to 2021. In individuals with premature ASCVD, we found that Lp(a) testing rates are higher with rates increasing from 2 % in 2017 to 11 % in 2021. The increased Lp(a) testing is likely multifactorial: (1) multi-society guidelines and organizations recommending testing for Lp(a) with personal or family history of ASCVD once with consideration of cascade testing in appropriate individuals [9], (2) ongoing Lp(a) clinical studies and Lp(a) reducing therapeutics being evaluated in clinical trials, (3) increased clinician awareness and education on Lp(a), (4) structured Prevention, Cardiometabolic, and Lipid Clinics aimed at optimizing ASCVD risk reduction using a variety of lifestyle interventions, pharmaceuticals, and interventions.

Our study found that cardiologists were most likely to order Lp(a). This reflects the need to raise awareness of the importance of Lp(a) as a cardiovascular risk factor in other specialties. While a study in a different academic center found neurology specialists to order Lp(a) tests most frequently, in our cohort this specialty did not drive significant Lp(a) testing despite the role of this biomarker in patients presenting with stroke [11]. Interestingly, we found that rheumatology specialists are increasingly ordering Lp(a) tests, which may represent the increasing awareness of the association between Lp(a) and pro-inflammatory and pro-thrombotic effects, in addition to pro-atherogenic effects, in development of cardiovascular disease.

Notably, while other similar studies have noted significant disparities in Lp(a) testing [8,17], our study was notable in that younger, Black and “other” race/ethnicity, and females were more likely to be tested. This should be interpreted with caution as this was a crude comparison without adjustment. If this is reflective of a true difference in practice, it is possible that the setting of JHH in the historically underserved community of East Baltimore has served to increase awareness of disparities around ASCVD risk reduction. 33 % of patients in our cohort had Lp(a) > 125 nmol/L and were more likely to be female and of Black race, which is consistent with studies showing differences in Lp(a) levels by race [18,19]. Our cohort has higher rates of elevated Lp(a), likely reflecting the population that was tested, which included more individuals of Black race and those with premature ASCVD. In individuals found to have elevated Lp(a), approximately one-fifth had a subsequent referral to cardiology or lipid clinic, and a similar percentage were initiated on lipid-lowering therapy, similar to other studies [20]. The median time to lipid-lowering therapy initiation of 318 days indicates that there is not only opportunity for greater initiation of evidence-based lipid medications, but more timely initiation.

In our subgroup analysis, those with premature ASCVD and elevated Lp(a) had higher rates of cardiology or lipid clinic referral but similar rates of lipid lowering medication prescription. The Johns Hopkins Ciccarone Advanced Lipid Disorders Clinic (JH Lipid Clinic) includes cardiology faculty with specialty training in lipid disorders in collaboration with coordinators, nurses, nurse practitioners, genetic counselors, specialty pharmacists, and dieticians to provide a comprehensive lipid management program. Before the first consultative visit the patient is required to complete a lipid panel (with LDL-C calculated by Martin/Hopkins equation) [[21], [22], [23]], apolipoprotein B, and lipoprotein(a). Once those labs are obtained the patient presents to the outpatient lipid clinic for a history, physical exam, and review of labs and imaging. The clinicians apply a validated set of criteria, the Dutch Lipid Clinic Network diagnostic criteria for Familial Hypercholesterolemia[[24], [25], [26], [27]] that categorizes patients by the likelihood of FH and the PREVENT calculator[28] for primary prevention. At the end of the first consultative visit the patient is typically clinically diagnosed with a lipid disorder and either intensified or initiated on lipid lowering therapy with a threshold LDL-C goal and/or triglyceride goal established.

The JH Lipid Clinic model is one approach on how to improve utilization of emerging biomarkers in developing personalized cardiovascular risk assessment in individuals at risk for or with cardiovascular disease. In addition, based on this study further work should study the role of clinical decision support within electronic medical records to encourage Lp(a) testing in those at increased risk of ASCVD events and based on levels refer patients to lipid management specialists who can aid in the process of management and determining need for cascade screening. A decision support intervention along with education has been associated with increased Lp(a) testing at other institutions [29,30]. Future work is also needed to develop educational interventions geared toward internal medicine providers.

Currently, it is under investigation if reducing Lp(a) levels leads to a reduction in adverse cardiovascular events. The ODYSSEY OUTCOMES and FOURIER trials showed that these medications reduce Lp(a) by approximately 20–30 % [31], however the associations of these reductions with reduced ASCVD risk were exploratory. In our cohort, we found that PCSK9 inhibitors accounted for more than half of new lipid lowering medications that were prescribed to patients after Lp(a) testing. An association between Lp(a) testing and PCSK9 inhibitor initiation has been reported in a large insurance claims database analysis in Germany [32]. Given the historically low rates of PCSK9 inhibitor utilization, Lp(a) testing may encourage utilization of this class of evidence-based medications, when indicated for LDL-C lowering, by allowing providers to personalize cardiovascular risk assessment for patients [33,34]. The HORIZON study (Assessing the Impact of Lipoprotein (a) Lowering With Pelacarsen [TQJ230] on Major Cardiovascular Events in Patients With CVD) will be the first study to assess whether reductions in Lp(a) through a monthly subcutaneous antisense oligonucleotide targeting apo (a) impacts cardiovascular outcomes, and will aid in determining how medication therapy should be tailored in patients with elevated Lp(a). In addition, other therapies, such as siRNA therapies and muvalaplin, that target Lp(a) lowering are in development and early trials.

Our study has several important limitations. Given this is a single institution study, the data may not be generalizable. The data was extracted retrospectively using ICD10 and CPT codes which may be subject to error. Similarly, we are not able to assess medication changes that may have occurred outside of the institution. In addition, we did not assess for family history of cardiovascular disease, despite there being guidelines to check Lp(a) in this population.

Based on single-center experience at a large academic medical center, the frequency of incident Lp(a) testing among patients with or with increased risk for ASCVD was low but increased significantly over 5 years. The increase in Lp(a) testing is encouraging and may be multifactorial in nature including new guideline recommendations, ongoing Lp(a) trials, increased education and clinician awareness, and structured referrals to specialty clinics, particularly our institution's lipid clinic. Moving forward, these findings support the need for population based Lp(a) testing strategies and additional evidence and guidance to support optimal clinical decision-making regarding risk management once Lp(a) elevation is diagnosed.

## Funding

Kaneka provided study funding but had no role in data collection, data analysis, manuscript review, writing, preparation, or the decision to submit this manuscript for publication.

## Disclosures

Outside of this work, under a license agreement between Corrie Health and the Johns Hopkins University, the University owns equity in Corrie Health and the University and Dr. Marvel and Dr. Martin are entitled to royalty distributions related to the Corrie technology. Additionally, Dr. Marvel and Dr. Martin are founders of and hold equity in Corrie Health. This arrangement has been reviewed and approved by the Johns Hopkins University in accordance with its conflict of interest policies.

Dr. Marvel has received funding for this work from Kaneka. Outside of this work, she has received material support from Apple and iHealth; has received funding from the Maryland Innovation Initiative, Wallace H. Coulter Translational Research Partnership, Louis B. Thalheimer Fund, PJ Schafer Cardiovascular Research Fund, the American Heart Association Empowered to Serve Business Accelerator.

Outside of this work, Dr. Martin has received material support from Apple and iHealth; has received funding from the Maryland Innovation Initiative, Wallace H. Coulter Translational Research Partnership, Louis B. Thalheimer Fund, the Johns Hopkins Individualized Health Initiative, the American Heart Association (20SFRN35380046, 20SFRN35490003, COVID19‐811,000, #878,924, and #882,415), the Patient‐Centered Outcomes Research Institute (ME‐2019C1‐15 328, IHS-2021C3–24,147), the National Institutes of Health (P01 HL108800 and R01AG071032), the David and June Trone Family Foundation, the Pollin Digital Innovation Fund, the PJ Schafer Cardiovascular Research Fund, Sandra and Larry Small, CASCADE FH, Google, Merck, and Amgen; has received personal fees for consulting from Amgen, AstraZeneca, Kaneka, NewAmsterdam, Novartis, Novo Nordisk, Sanofi, and 89bio

**Figure:**
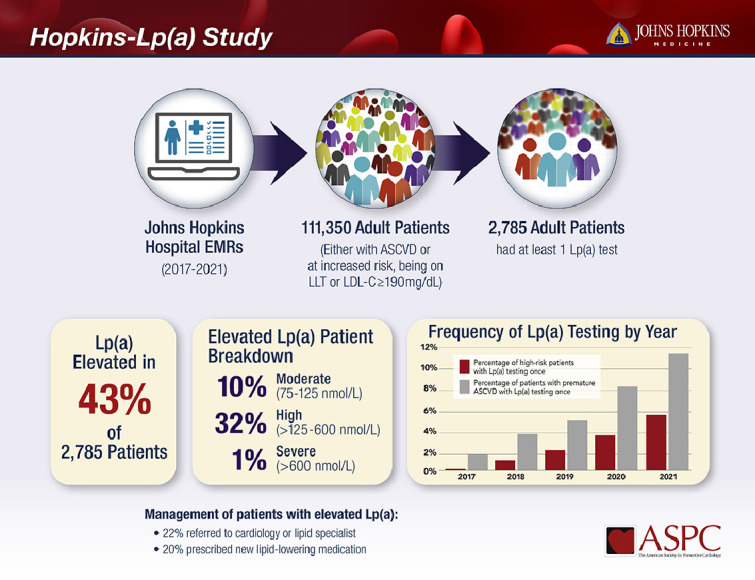
Central illustration

## CRediT authorship contribution statement

**Yehuda Eidensohn:** Writing – original draft, Project administration, Methodology, Formal analysis, Data curation, Conceptualization. **Anjali Bhatla:** Writing – original draft, Formal analysis, Data curation, Conceptualization. **Jie Ding:** Formal analysis, Data curation, Conceptualization. **Roger S. Blumenthal:** Writing – review & editing. **Seth S. Martin:** Writing – review & editing, Supervision, Resources. **Francoise A. Marvel:** Writing – review & editing, Supervision, Project administration, Methodology, Funding acquisition, Conceptualization.

## Declaration of competing interest

The authors declare the following financial interests/personal relationships which may be considered as potential competing interests:

Francoise Marvel reports financial support was provided by Kaneka Corporation. Francoise Marvel reports a relationship with Corrie Health that includes: equity or stocks. Francoise Marvel, Seth Martin reports a relationship with Apple Inc that includes: funding grants. Francoise Marvel, Seth Martin reports a relationship with Maryland Innovation Initiative that includes: funding grants. Francoise Marvel, Seth Martin reports a relationship with Wallace H. Coulter Translational Research Partnership that includes: funding grants. Francoise Marvel reports a relationship with Louis B. Thalheimer Fund, Seth S. Martin that includes: funding grants. Francoise Marvel, Seth Martin reports a relationship with PJ Schafer Cardiovascular Research Fund that includes: funding grants. Francoise Marvel reports a relationship with American Heart Association Empowered to Serve Business Accelerator that includes: funding grants. Seth Martin reports a relationship with Johns Hopkins Individualized Health Initiative that includes: funding grants. Seth Martin reports a relationship with American Heart Association that includes: funding grants. Seth Martin reports a relationship with Patient-Centered Outcomes Research Institute that includes: funding grants. Seth Martin reports a relationship with National Institutes of Health that includes: funding grants. Seth Martin reports a relationship with David and June Trone Family Foundation that includes: funding grants. Seth Martin reports a relationship with Pollin Digital Innovation Fund that includes: funding grants. Seth Martin reports a relationship with Sandra and Larry Small that includes: funding grants. Seth Martin reports a relationship with CASCADE FH that includes: funding grants. Seth Martin reports a relationship with Google Inc that includes: funding grants. Seth Martin reports a relationship with Merck that includes: funding grants. Seth Martin reports a relationship with Amgen Inc that includes: consulting or advisory and funding grants. Seth Martin reports a relationship with AstraZeneca that includes: consulting or advisory. Seth Martin reports a relationship with Kaneka Corporation that includes: consulting or advisory. Seth Martin reports a relationship with NewAmsterdam Pharma Corporation that includes: consulting or advisory. Seth Martin reports a relationship with Novartis that includes: consulting or advisory. Seth Martin reports a relationship with Novo Nordisk that includes: consulting or advisory. Seth Martin reports a relationship with Sanofi that includes: consulting or advisory. Seth Martin reports a relationship with 89bio Inc that includes: consulting or advisory. If there are other authors, they declare that they have no known competing financial interests or personal relationships that could have appeared to influence the work reported in this paper.
